# Effects of precipitation and clipping intensity on net primary productivity and composition of a *Leymus chinensis* temperate grassland steppe

**DOI:** 10.1371/journal.pone.0190450

**Published:** 2017-12-29

**Authors:** Feng He, Kun Wang, David B. Hannaway, Xianglin Li

**Affiliations:** 1 Institute of Animal Science, Chinese Academy of Agricultural Sciences, Beijing, People's Republic of China; 2 Department of Grassland Science, China Agricultural University, Beijing, People's Republic of China; 3 Department of Crop & Soil Science, Oregon State University, Corvallis, Oregon, United States of America; Pacific Northwest National Laboratory, UNITED STATES

## Abstract

*Leymus chinensis* (Trin.) is the dominant vegetation type in eastern Eurasian temperate grasslands but is decreasing due to the combined pressure of reduced precipitation and overgrazing. This study evaluated the separate and combined effects of precipitation and defoliation on net primary productivity (NPP) and composition of a *L*. *chinensis* steppe to promote the sustainable development of temperate grasslands through improved management practices. The effects of three precipitation gradients (precipitation unchanged, reduced by 50%, and increased by 50%) and two clipping intensities (clipping once or twice per year) were examined on NPP and composition of the *L*. *chinensis* community using a 7-year *in situ* controlled trial at the Guyuan State Key Monitoring and Research Station of Grassland Ecosystem in China. The results showed that: (1) a 50% reduction in natural precipitation significantly decreased NPP; a 50% increase in precipitation did not significantly increase NPP, but it decreased the importance value of *L*. *chinensis* because more water promoted the growth of competing species. (2) Clipping twice per year increased NPP, but the increase was from the dry matter of other species (DMO) component, and not from the dry matter of *L*. *chinensis*. (3) The standardized coefficients of a regression model (β) for DMO, NPP, and the importance value of *L*. *chinensis* were 0.685, 0.532, and −0.608 for precipitation, and 0.369, 0.419, and −0.276 for clipping mode, respectively. This study demonstrated that variation in precipitationis the key driver of NPP and composition of a *L*. *chinensis* steppe under the precipitation range and clipping intensities evaluated. This improved understanding of the effects of precipitation and clipping on NPP and composition will allow for improved, sustainable management of *L*. *chinensis* temperate grassland steppes.

## Introduction

Temperate grassland is an important vegetation type in arid and semiarid regions around the world. These grassland harbor diverse plant, animal, and insect species, and numerous ecosystem services including wildlife habitat, forage for domestic and wild ruminants, water purification, increased soil organic matter, and carbon sequestration [1]. The Eurasian Steppe is the largest temperate grassland in the world, extending over 8000 km from north-eastern China to Hungary [2]. Although many studies have focused on temperate grassland, it is difficult to predict its responses to climate change because of the erratic annual and seasonal precipitation patterns, and the complicating nature of the previous year’s growth of perennial species influencing current-year growth [1,3–9]. Nevertheless, most grassland response variables are more sensitive to changes in precipitation than to other climatic factors [10–15].

*Leymus chinensis* (Trin.) Tzvel is a native, perennial, rhizomatous grass. Grasslands dominated by L. *chinensis* are widely distributed and it is the dominant vegetation type in eastern Eurasian temperate grasslands. This grassland type occupies an area of 3 × 10^5^ ha in Inner Mongolia, and it plays a key role in providing livestock forage, wildlife habitat, and environmental security to the nation [16,17]. However, these grasslands have been degrading since the 1980s under the combined pressure of climate change and human disturbance [18–21]. The *L*. *chinensis* steppe is a dynamic system that is strongly affected by clipping and precipitation, primary drivers of plant community composition and net primary productivity (NPP) [22]. Thus, a large body of research has focused on the relationship between NPP and precipitation. Ni found a significant positive relationship between NPP and annual and summer precipitation in temperate northern China [23]. Bai et al. found that precipitation on the *L*. *chinensis* steppe during January to July was a significant variable in simplified multiple regression analyses [24]. Baoyin et al. reported that a quadratic function was a better predictor of NPP than linear or logarithmic equations [25]. Both quantity and timing of precipitation play a key role in the productivity and species richness of annual and perennial species in temperate grassland ecosystems [22,26].

Clipping *L*. *chinensis* to produce hay is a normal practice in temperate grasslands [27–29]. However, since the 1980s, there have been many reports that clipping reduces NPP [28–32]. The practice of clipping once yearly gave the highest NPP in a 27-year experiment, as compared with other treatments [30]. In contrast, Schiborra et al. found that multiple clipping treatments gave higher NPP in a 1-year cutting trial [33]. Previous studies have focused on the effects of clipping regime (frequency) on species composition, diversity, and NPP. Different growth-form groups were found to respond differently to clipping intensity. The biomass of short bunchgrasses and annual and biannual species increased, whereas the biomass of perennial rhizome grasses such as *L*. *chinensis* decreased [25].

Clipping and precipitation are important factors in controlling dynamic changes of temperate grasslands [34,35]. Thus, the changes in precipitation predicted by various in climate change models, especially in conjunction with increased clipping pressure, might have long-term impacts on the sustainability of temperate grassland ecosystems. However, a broad understanding of the responses of temperate grassland to precipitation and clipping remains elusive [1,25], since it is difficult to evaluate separately the effects of precipitation versus clipping on *L*. *chinensis* grasslands.

The objective of this study was to simultaneously evaluate the effects of precipitation and clipping frequency on NPP and species composition in a *L*. *chinensis* temperate grassland. To achieve this, rain-out shelters and irrigation were used to manage the amount of precipitation on a *L*. *chinensis* steppe *in situ*. This allowed us to clarify the individual effects of precipitation and clipping on a *L*. *chinensis* temperate grassland.

## Materials and methods

### Experimental site

The study was conducted at the Guyuan State Key Monitoring and Research Station of Grassland Ecosystem in China (41°46′N, 115°40′E, 1400 m a.s.l.), located at the southern edge of the Xilingol steppe grassland in Hebei Province, northern China. This region has a semiarid continental climate with mean annual precipitation of 350 mm, primarily distributed between May and September. The mean annual temperature is 1.0°C and the mean minimum temperature in the coldest month (January) is -17.4°C. The growing season is approximately 100 days long, from May to September [36]. The major soil type is sandy loam dark chestnut soil (Calcic-orthic Aridisol in the US classification system) [37], and is slightly alkaline (pH 7.6). The experiment was conducted within a fenced, permanent 2-ha research paddock of a typical temperate grassland dominated by *L*. *chinensis*. Additional species included *Cleistogenes squarrosa* (Trin.) Keng (a short perennial bunchgrass) and the forb *Artemisia eriopoda* Bunge.

### Treatments and experimental design

**Fig 1:**
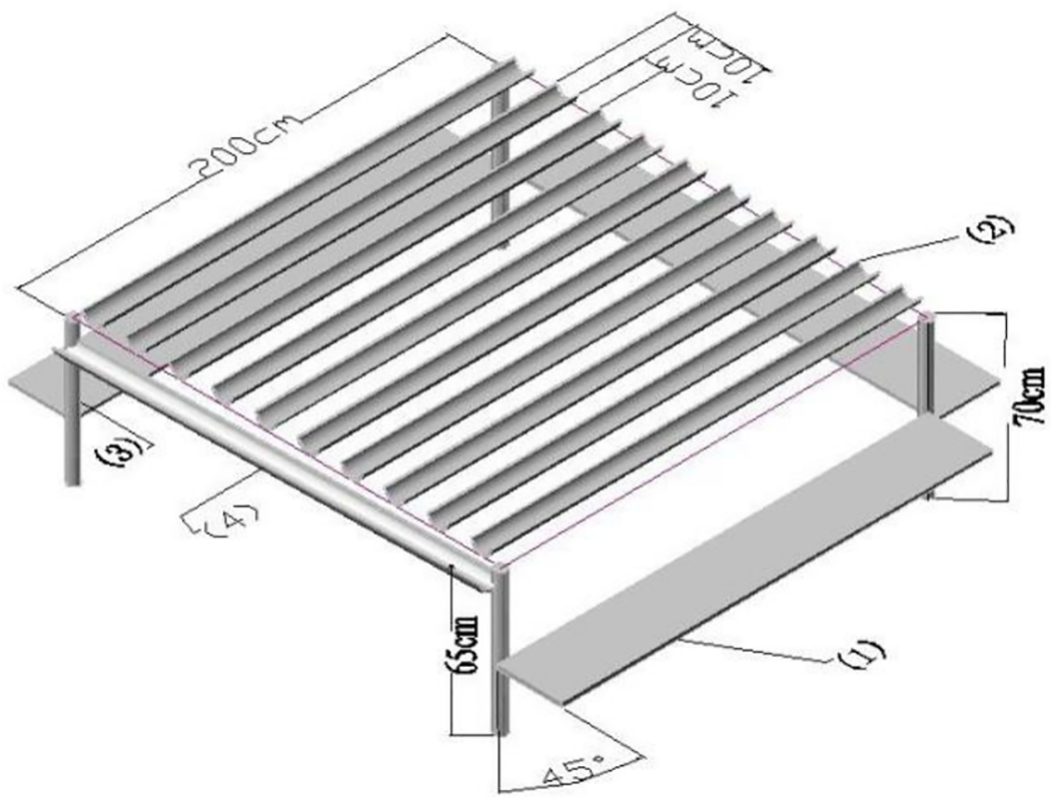
Transparent rain-out shelter. (1) Side shelter made of transparent plastic film, length 200 cm, width 30 cm, braced to maintain a 45° angle; (2) Shelter above made by a splitting transparent polyvinyl chloride (PVC) tubing lengthwise, length 200 cm, width 10 cm, depth 4.8 cm, distance 10 cm, fixed on a removable wooden shelf; (3) Four corner braces, and (4) Side water guide tube made of split PVC tubing, fixed at 60 cm on the front brace and at 55 cm on the rear brace to form a slope for drainage.

**Fig 2:**
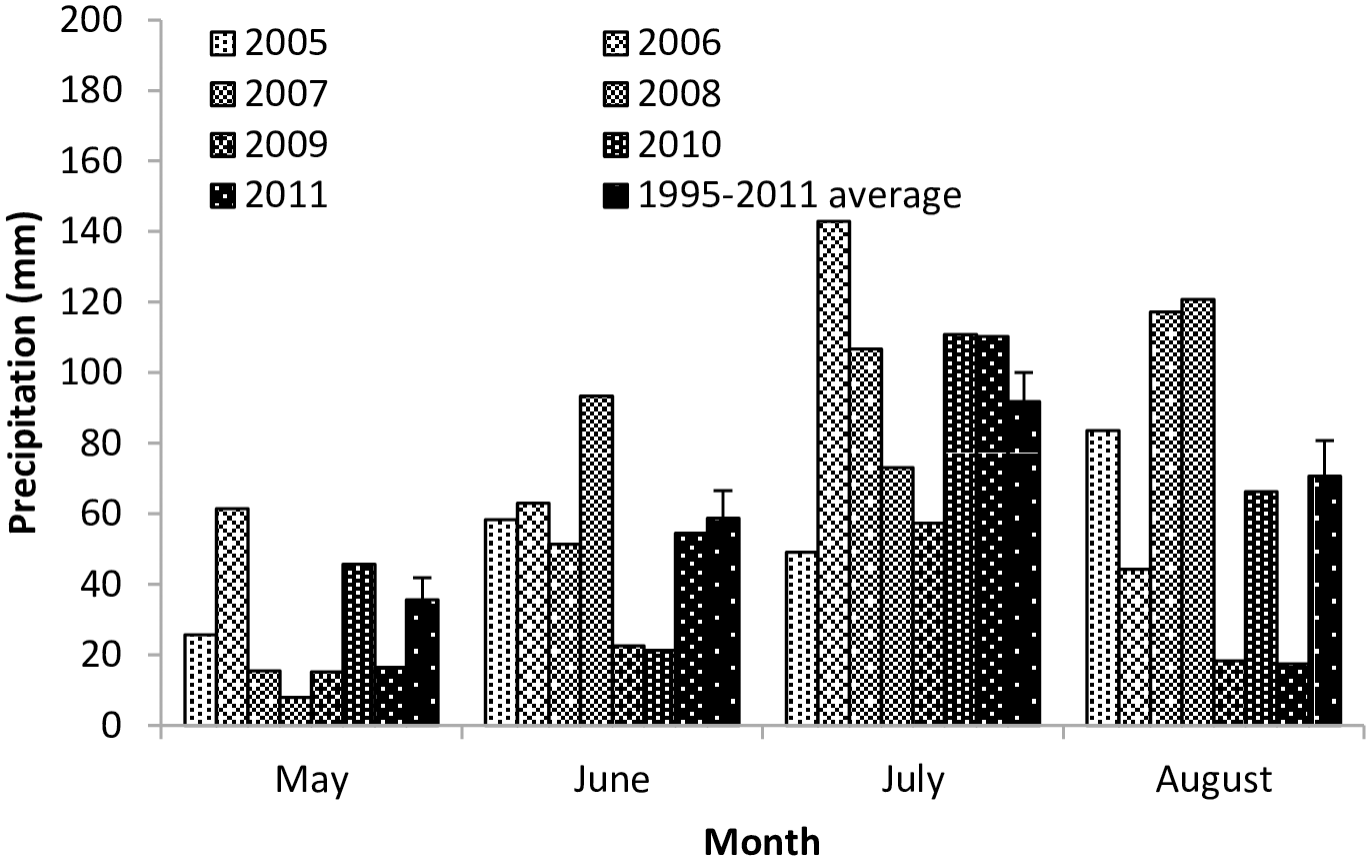
Growing season rainfall distribution for 2005 through 2011. Black bars indicate the regional monthly averages (mean + SE) from 1995 to 2011.

Six experimental treatments included three precipitation levels and two clipping regimes. The precipitation treatments were as follows: (1) 50% reduction of natural precipitation by using permanent rain-out shelters (Fig 1); (2) unmodified ambient precipitation; and (3) 50% increase of precipitation by irrigation immediately after each rainfall event. These were designated water reduction, ambient, and water addition treatments, respectively. The water reduction treatment was targeted to be 50% of natural rainfall. Plot actual precipitation (PAP) was measured by two rain gauges under each transparent rain-out shelter during the growing season of 2005. Sometimes a little more rain fell within the plot and sometimes a little less due to the influence of wind during rainfall events. However the total PAP in the water reduction treatment was approximately half that of natural rainfall during the growing season. PAP in the ambient treatment was naturally occurring rainfall, and that in the water addition treatment was 1.5 times the amount of ambient, which was achieved by increasing precipitation by 50% using irrigation according to the rain gauge value.

The two clipping regimes were as follows: (1) clipping once each year, during the third week of August; and (2) clipping twice each year, first at the end of June and second during the third week of August. These were designated C1 and C2. These six treatments (3 precipitation gradients and 2 clipping regimes) were arranged in a randomized complete block design, with three replications. Each plot was 2 × 2 m. To control runoff and percolation, water reduction and water addition plots were encircled by a high-density polyethylene impermeable membrane (0.5 mm), which extended 57 cm below the soil surface and 3 cm above it. To minimize the shadow effect on plant photosynthesis, the thickness of transparent polyvinyl chloride (PVC) tube was 3 mm and light transmittance was 85%. The rain-out shelter was designed so that it was easy to remove on sunny days by detaching it from the corner braces. The rain-out shelters were stored in a warehouse from September to April.

### Data collection

Precipitation events during the growing season (May to August) from 2005 through 2011 were recorded with a simple rain gauge located at the experimental site. The regional monthly average precipitation data for 1995–2011 were obtained from Guyuan Ranch Meteorological Station located 30 km from the experimental site.

NPP was measured each year from 2005 through 2011 (except 2008) at the end of June for C2 treatments and in late August for both C1 and C2 treatments. Each plot’s NPP was determined species-by-species by harvesting a 1 × 1 m quadrat of in the center of the plot to a 2 cm residual height. This results in obtaining more than 90% of the biomass obtained by clipping to ground level. After sampling, the entire plot was clipped to the same height. All plant samples were oven-dried at 80°C and weighed. NPP was separated into two parts, *L*. *chinensis* dry matter (DML) and other-species dry matter (DMO). The importance value of *L*. *chinensis* (IVL) was calculated as the percentage of total NPP: IVL = (*L*. *chinensis* dry matter/NPP)×100%. The PAP, DML, DMO, NPP, and IVL original data were recorded from 2005 to 2011 (S1 File).

### Data analysis

Monthly and seasonal precipitation was measured for 2005–2011 and the coefficient of variation (CV) was calculated: CV = (standard deviation/mean) × 100.

A general linear model (GLM) was used to test the main and interactive effects of precipitation, clipping, and year on community composition (DML, DMP, and NPP), and community structure (IVL) using the multivariate module of SPSS (version 19.0, SPSS Inc., Chicago, Illinois, USA, 2004). Precipitation, clipping, and year were taken as fixed factors. When the effects of precipitation and clipping were significant, means were separated using the least significant difference (LSD) test. The results of statistical analyses were graphed using Microsoft Excel software (Microsoft, Seattle, Washington, USA, 2010).

The regression models for modeling the relationship between dependent variables (NPP, DML, DMO, IVL) and independent variables (precipitation gradient, clipping intensity) were built using the linear module of SPSS. The 6-year mean NPP, DML, DMO, and IVL value of each plot was used as the dependent variable, while precipitation and clipping were used as the independent variables. The precipitation gradient consisted of water reduction, ambient, and water addition as described above. The clipping regimes were C_1_ and C_2_. The standardized regression coefficient (beta) of precipitation gradient and clipping frequency in each linear regression model of NPP, DML, DMO, and IVL was evaluated by t- test.

### Ethics statement

All observational and field studies at the experimental site were undertaken with relevant permissions from the owners–Guyuan station, China Agricultural University. The location is not privately-owned or protected in any way and the field studies did not involve endangered or protected species.

## Results

### Characteristics of rainfall during growing season

From 2005 to 2011, the average rainfall in the growing season (May through August) was 239 mm. Minimum rainfall was 113.5 mm in 2009, and the maximum was 311.8 mm in 2006 (CV, 29.15%) (Fig 2). The 7-year average for May rainfall was 26.8 mm (CV, 72.77%). The average rainfall was 52.1 mm (CV, 47.61%) in June, 92.9 mm (CV, 36.48%) in July, and 66.8 mm (CV, 64.16%) in August.

### Effect of precipitation and clipping on NPP, DML, DMO, and IVL

The DML was 100.5 and 87.2 g m^-2^ in ambient and water reduction treatments, respectively, both of which were greater than that of the water addition treatment (76.6 g m^-2^; *p* < 0.05). The DMO significantly (*p* < 0.05) increased with increasing precipitation, with values of 43.6, 66.4, and 85.1 g m^-2^ in the water addition, ambient, and water reduction treatments, respectively. There were significant differences between precipitation treatments (R) for *NPP*. *NPP* of water reduction was 130.8 g m^-2^, substantially lower than those in ambient and water addition treatments (166.9 and 161.8 g m^-2^, respectively; *p* < 0.05). There was no significant difference of NPP between ambient and water addition. The IVL significantly decreased with increasing precipitation; it was 60.5% and 67.0% in the ambient and water reduction treatments, respectively, and significantly lower (48.1%) in the water addition treatment (*p* < 0.05). There was no significant difference in IVL between ambient and water reduction treatments (Tables 1 and 2).

**Table 1:** Results (*p*-values) of general linear model on the effects of precipitation (R), clipping (C), year (Y), and their interactions on the dry matter of dominant species, net primary productivity (NPP) and importance value of *Leymus chinensis* (IVL).

Sources of Variation	df	DML	DMO	NPP	IVL
R	2	**< 0.01**	**< 0.01**	**< 0.01**	**< 0.01**
C	1	0.77	**< 0.01**	**< 0.01**	**0.02**
Y	5	**< 0.01**	**< 0.01**	**< 0.01**	**< 0.01**
R*C	2	**0.05**	0.37	0.41	0.38
R*Y	10	0.52	0.29	**< 0.01**	0.80
C*Y	5	0.09	0.07	0.40	**0.03**
R*C*Y	10	0.94	0.99	0.78	0.98

Abbreviations: DML, dry matter of *Leymus chinensis*; DMO, dry matter of other species. Note: Bold font indicates significance at *p* < 0.05.

**Table 2:** Effect of precipitation and clipping on composition and net primary productivity (NPP) of *L*. *chinensis* grassland from 2005 through 2011.

Precipitation treatments	Clipping regimes	DML (g m^-2^)	DMO (g m^-2^)	NPP (g m^-2^)	IVL (%)
R_1_	Both	87.2 ± 5.5 a	43.6 ± 5.0 a	130.8 ± 5.6 b	67.0 ± 2.7 a
R_2_	Both	100.5 ± 5.5 a	66.4 ± 5.0 b	166.9 ± 5.6 a	60.5 ± 2.7 a
R_3_	Both	76.6 ± 5.5 b	85.1 ± 5.0 c	161.8 ± 5.6 a	48.1 ± 2.7 b
All	C_1_	87.3 ± 4.6	55.9 ± 4.5 b	143.2 ± 4.8 b	62.0 ± 2.4 a
All	C_2_	88.9 ± 4.6	74.2 ± 4.5 a	163.1 ± 4.8 a	55.0 ± 2.4 b

Abbreviations: DML, dry matter of *Leymus chinensis*; DMO, dry matter of other species; IVL, importance value of *L*. *chinensis*; R_1_, 50% reduction of natural precipitation achieved using permanent rain-out shelters (Fig 1); R_2_, ambient precipitation; R_3_, 50% increase in precipitation by irrigating immediately after each rainfall event; C_1_, clipping once per year in third week of August; C_2_, clipping twice per year; at end of June and in third week of August. Note: Values are means ± SE of three replications. Within each column, different letters indicate significant difference at *p* < 0.05.

There was no significant difference in DML between clipping regimes. The mean DML from 2005 through 2011 were 87.3 and 88.9 g m^-2^ for C1 and C2, respectively. For DMO and NPP, dry matter increased significantly with clipping intensity. The mean DMO was 55.9 g m^-2^ for C1, increasing to 74.2 g m^-2^ for C2, an increase of 18.3 g m^-2^. The mean NPP was 143.2 g m^-2^ for C1 and 163.1 g m^-2^ in the C2 treatment, an increase of 19.9 g m^-2^. IVL decreased significantly with increased clipping intensity; 62.0% for C1, decreasing 7% to 55.0% in C2 (Tables 1 and 2).

The values of DML, DMO, NPP, and IVL differed significantly among years (Table 1). An interactive effect between precipitation and clipping treatments was detected for DML. The effects of precipitation on NPP also varied among years (Table 1). The NPP in the water addition treatment was higher than that in other treatments in 2007 and 2011, but lower than that in other treatments in the other years. The effects of clipping on IVL varied among years (Table 1). The IVL was higher with increased clipping frequency in 2006 and 2009 but lower with increased clipping frequency in the other years.

Regression model of DML, DMO, NPP, and IVL for precipitation gradients and clipping regimes The adjusted r^2^ of the DML regression model was—0.082 (*p* = 0.705), indicating no statistical significance. The adjusted r^2^ values of the regression models for DMO, NPP and IVL were 0.552, 0.387, and 0.372, respectively (statistically significant at *p* < 0.05) (Table 3).

**Table 3:** Regression models for *dry matter of L*. *chinensis* (DML), dry matter of other species (DMO), net primary productivity (NPP), and importance value of *L*. *chinensis* (IVL) for precipitation gradients and clipping regimes.

Dependent variable	Regression model	Adjusted r^2^	F value
DML	Y_1_ = 96.248–10.585X_1_+1.626X_2_	- 0.082 ^ns^	0.358
DMO	Y_2_ = --3.906+41.531X_1_+18.283X_2_	0.552**	11.490
NPP	Y_3_ = 92.342+30.946X_1_+19.907X_2_	0.387**	6.366
IVL	Y_4_ = 87.935–18.905 X_1_-7.002X_2_	0.372*	6.045

Y_1_, Dependent variable DML; Y_2_, dependent variable DMO; Y_3_, dependent variable NPP; Y_4_, dependent variable IVL; X_1_, precipitation gradient; X_2_, clipping regime.

* Significance at p < 0.05 level;

** significance at *p* < 0.01 level;

^NS^ not significant.

From the non-standardized coefficient (beta) of the regression models, DMO increased with increasing precipitation gradient and clipping regime, similar to the NPP regression model. In contrast, IVL decreased with increasing precipitation gradients and clipping intensity (Table 3).

To compare the standardized effect of precipitation and clipping intensity on DML, DMO, NPP and IVL, we further tested beta value for these parameters in the regression model. Neither **precipitation gradient** nor **clipping regime** was statistically significant in the DML regression model. In the DMO regression model beta values were 0.685 and 0.369, respectively, both significant at *p* < 0.05. In the NPP regression model, beta values for **precipitation gradient** and **clipping regime** were 0.532 and 0.419, respectively, both significant at *p* < 0.05. In the IVL regression model beta values for **precipitation gradient** and **clipping regime** were −0.608 and −0.276, with significance at *p* < 0.01 and no significance, respectively (Table 4).

**Table 4:** Standardized coefficient of regression model of dry matter of *Leymus chinensis* (DML), dry matter of other species (DMO), net primary productivity (NPP), importance value of L. chinensis (IVL) for independent variable precipitation gradient (R) and clipping regime (C).

Independent variable	DML		DMO		NPP		IVL	
	beta	t value	beta	t value	beta	t value	beta	t value
R	-0.210^ns^	-0.832	0.685**	4.220	0.532*	2.803	-0.608**	-3.167
C	0.039^ns^	0.157	0.369*	2.275	0.419*	2.208	-0.276^ns^	-1.436

beta, standardized coefficient of regression model.

* Significance at *p* < 0.05 level;

** significance at *p* < 0.01 level;

^NS^ not significant.

## Discussion

### Precipitation and NPP

The growing-season and monthly precipitation varied greatly among years, similar to data from an adjacent experiment station, the Inner Mongolia Grassland Ecosystem Research Station (IMGERS), where they reported a CV of annual precipitation from 1998 to 2012 of 22% [38]. With this amount of precipitation variation, Bai *et al*. found that January–July precipitation was a significant variable in a simplified multiple regression equation for predicting NPP (r^2^ = 0.25, *p* < 0.01) from long-term experimental field data [24].

A previous study reported that NPP declined when precipitation is above 380 to 420 mm [30]. This decline in NPP under heavier precipitation may be attributed to reduced solar radiation and lower temperature accompanying precipitation [30]. In this study, we observed the competition between dominate species *L*. *chinensis* and other species influences the relationship between NPP and precipitation. From late July, *L*. *chinensis* begins to produce buds and new shoots before entering a dormant stage [39]. At this stage, photosynthetic products are transported to propagative organs and not partitioned to dry matter accumulation. However, some other species, especially the C_4_ plant *C*. *squarrosa* and the forb *A*. *eriopoda* enter a rapid growth period with increased precipitation. This resulted in an increased DMO with increased precipitation. This agrees with previous research of Ni, who found that the percentage of forbs increased with precipitation [23]. Due to the increase in DMO with increasing precipitation, the IVL of *L*. *chinensis* decreased with increasing precipitation. Therefore, the dominant species *L*. *chinensis* was restricted by other species, which limited the increase in its NPP.

### Clipping and NPP

Many researchers have studied “reasonable and sustainable” clipping intensities over a long period [27,29,40]. Our results showed that NPP in the *L*. *chinensis* grassland increased with more frequent clipping. This is consistent with the findings of Schiborra *et al*., who found that multiple cuts led to higher NPP due to the compensatory growth effect [33]. However, our findings are inconsistent with the results of Baoyin *et al*. [30]. This may be due to species differences, because the response of dry matter accumulation after defoliation differs among species. Tall grasses tend to show decreased NPP after defoliation, while short grasses and annual and biennial species benefit from frequent defoliation [25]. In our research, DML was not affected by the clipping regime. However, the DMO was significantly greater with two clippings per year than with one clipping per year, and total biomass was also significantly greater with higher clipping frequency. Because the higher clipping intensity increased DMO but did not change DML, the IVL also decreased. Thus, the status of *L*. *chinensis* in the community was degraded with increased clipping intensity.

### Dominant role of precipitation in NPP

Precipitation is a critical determinant of the NPP of temperate grassland ecosystems [3,41–44]. Our results (beta values of regression models for precipitation gradient and clipping frequency) showed that the growing season precipitation was more important than the clipping frequency for DMO, NPP, and IVL (Table 4). Precipitation is the dominant factor for NPP and the composition of the *L*. *chinensis* steppe. This is in agreement with research conducted in arid and semiarid ecosystems that community structure tends to be responsive to short-term rainfall fluctuations [45], and stochastic fluctuations in precipitation have stronger effects than intensity of use on community structure [46]. The effect of precipitation on NPP is not only determined by its amount but also by its seasonal pattern and its history in previous years [3,32,47]. This makes it difficult to clarify the contribution of precipitation to NPP. In addition, competition among species also affect the relationship between precipitation and NPP. This illustrates the need for further research on the effects of precipitation at the species and functional group levels.

The results of our study showed that growing season precipitation is the dominant factor determining the variation of NPP in a *L*. *chinensis* steppe. There are extreme variations in precipitation in these regions; therefore, there is considerable uncertainty regarding the forage supply to livestock in the *L*. *chinensis* steppe region. In China, one of most important strategies for promoting sustainable grassland development is balancing forage supply and livestock forage requirements to avoid the risks of over-use. The results of this study highlight the importance of assessing the biological capacity of the region to develop rational grazing management strategies. This includes understanding vegetation dynamics driven by the amount and seasonal distribution of precipitation.

## Conclusions

Our results show that the primary driver for the NPP of *Leymus chinensis*-dominated short grasslands (steppe) is limited precipitation. Clipping twice per year increased NPP. However, the increase in production was not from the dominant species (*L*. *chinensis*) but from other grasses and forbs that benefited from the removal of the taller species. The interaction between precipitation and clipping intensity was significant for DML. Our results suggest that the sustainable development of *L*. *chinensis* temperate grasslands requires not only control of utilization intensity, but also a knowledge of the processes and mechanisms of vegetation dynamics in response to variations in precipitation.

## Supporting information

S1 File Plot actual precipitation, dry matter and importance value of *Leymus chinensis* data from 2005 to 2011. (XLSX)
